# The Impact of Centralization of Obstetric Care Resources in Japan on the Perinatal Mortality Rate

**DOI:** 10.1155/2013/709616

**Published:** 2013-09-18

**Authors:** Akira Sudo, Yoshiki Kuroda

**Affiliations:** Department of Public Health, Faculty of Medicine, University of Miyazaki, 5200 Kihara, Kiyotake-cho, Miyazaki City, Miyazaki 889-1692, Japan

## Abstract

*Objective*. We investigated the effects of the centralization of obstetricians and obstetric care facilities on the perinatal mortality rate in Japan. *Methods*. We used the Gini coefficient as an index to represent the centralization of obstetricians and obstetric care facilities. The Gini coefficients were calculated for the number of obstetricians and obstetric care facilities of 47 prefectures using secondary medical care zones as units. To measure the effects of the centralization of obstetricians and obstetric care facilities on the outcomes (perinatal mortality rates), we performed multiple regression analysis using the perinatal mortality rate as the dependent variable. *Results*. Obstetric care facilities were more evenly distributed than obstetricians. The perinatal mortality rate was found to be significantly negatively correlated with the number of obstetricians per capita and the Gini coefficient of obstetric care facilities. The latter had a slightly stronger effect on the perinatal mortality rate. *Conclusion*. The centralization of obstetric care facilities can improve the perinatal mortality rate, even when increasing the number of obstetricians is difficult.

## 1. Background and Objective

In Japan, obstetric care facilities and the number of obstetricians are decreasing [1]. One of the reasons for this could be the risks in delivery system, and sometime lawsuits could be held between obstetrician and pregnant women. And the other is that obstetricians usually are working for a long time. It is debatable whether this shortage can be attributed to the decrease in the absolute number of obstetricians or a maldistribution. A theory proposed by Newhouse JP insisted that an increase in the number of obstetricians will result in the uniformity of their distribution [2].

The Japanese Ministry of Health, Labor, and Welfare, the Japan Medical Association, and the Japan Society of Obstetrics and Gynecology recommended the centralization of obstetric care facilities [3]. As a result, many small obstetric care facilities have been closed in the rural areas. Centralization is expected to increase the number of medical facilities with multiple full-time obstetricians and ensure safe childbearing care. Previous studies have shown that obstetric care outcomes are favorable in communities with large obstetric care facilities [4, 5]. On the other hand, the closure of nearby medical facilities due to centralization is expected to result in pregnant women having to travel longer distances to seek the care they need in the event of an emergency [6]. Thus, the centralization of obstetric care facilities could make access to such facilities difficult for some residents. In a previous study, it was shown that pregnant women who live in rural areas experience more difficulty in accessing medical care than those living in urban areas [7]. It has also been shown that poor access to obstetric care facilities is associated with poor outcomes [8–12]. Thus, the obstetrician shortage is accompanied by a raging debate as to whether centralization is a useful measure.

To the best of our knowledge, no study has evaluated the effects of the number and centralization of obstetricians and obstetric care facilities, respectively, on specific outcomes. The objective of the present study is to examine the effects of the centralization of obstetricians and obstetric care facilities on the perinatal mortality rate in Japan.

## 2. Materials and Methods

We used the Gini coefficient to evaluate the effect of the centralization of obstetricians and obstetric care facilities. The Gini coefficient, an economical concept developed by the Italian statistician Gini C. in 1936, represents the uniformity of incomes [13, 14]. The Gini coefficient is also applied to the studies related to medical care. Several previous studies have used it to evaluate the distribution of medical care resources [15–24]. Data from a health facility census conducted by the Ministry of Health, Labor, and Welfare [1] (concerning the numbers of obstetricians and obstetric care facilities) and a national census conducted by the Ministry of Internal Affairs and Communications [25] (concerning municipality populations) were used to calculate the Gini coefficients in the present study.

Secondary medical care zones (SMCZs) are decided according to the Medical Service Law. An SMCZ is determined by the proportional level of medical care provided for situations requiring stays in hospitals and other facilities in a certain area. In addition to a community's population and area, many other factors are taken into account, including geographic and other natural conditions, fulfillment of demands in daily life, and social conditions such as transportation. Japan comprises 47 prefectures, each of which is divided, based on size, into a number of SMCZs. There are a total of 348 SMCZs, each of which comprises multiple municipalities.

The Gini coefficients were calculated using a Lorenz curve. In order to determine the Lorenz curve, the numbers of obstetricians and obstetric care facilities per SMCZ within each prefecture were calculated. These numbers were then arranged in an ascending order. The cumulative relative frequencies of SMCZs were plotted on the *x*-axis, while the respective cumulative relative frequencies of the number of obstetricians and obstetric care facilities were plotted on the *y*-axis. The line connecting the starting point and all other points is the Lorenz curve. The area between the Lorenz curve and the 45° line (also known as the line of perfect equality), multiplied by 2, is called the Gini coefficient. The values of the Gini coefficient range from 0 to 1. The Lorenz curve approaches a 45° line when the distribution is equal and shifts away from it, to the lower right, when it is unequal. A Gini coefficient close to 0 represents a small disparity and that close to 1 a large disparity (i.e., a more unequal distribution).

We then calculated the Gini coefficients of obstetricians and obstetric care facilities in all prefectures. In order to investigate the effects of centralization of obstetricians and obstetric care facilities, we used perinatal mortality rates, obtained from the population dynamics survey conducted by the Ministry of Health, Labor and Welfare between 2006 and 2010 [26], as the outcome. Dependent variables included the total fertility rate (also sourced from the previously mentioned population dynamics survey [26]), per capita numbers of obstetricians and obstetric care facilities for all prefectures (sourced from the census of health care facilities conducted by the Ministry of Health, Labor, and Welfare in 2008 [1] and the national census conducted by the Ministry of Internal Affairs and Communications in 2010 [25]), and the calculated Gini coefficients for obstetricians and obstetric care facilities in all prefectures.

We performed multiple regression analyses in order to examine the effects of the centralization of obstetricians and obstetric care resources on the outcomes. Statistical analysis was performed with SPSS for Windows, version 17.0, with the level of statistical significance set at *P* < 0.05.

## 3. Results

The Gini coefficients of per capita numbers of obstetricians and obstetric care facilities for all prefectures are shown in Table 1. Large disparities in the Gini coefficients of obstetricians and obstetric care facilities were observed among prefectures (0.051–0.382 for obstetric care facilities and 0.076–0.513 for obstetricians). The Gini coefficients for obstetricians tended to exceed those for obstetric care facilities. 

The results of the multiple regression analysis about the effects of the centralization of obstetricians and obstetric care facilities on the perinatal mortality rate are shown in Table 2. A significant negative correlation was observed between the perinatal mortality rate and the per capita number of obstetricians. An increase in the number of obstetricians was shown to result in a decrease in the perinatal mortality rate. In addition, a significant negative correlation was observed between the perinatal mortality rate and the Gini coefficient of obstetric care facilities. Increasing centralization of obstetric care facilities was shown to result in a decrease in the perinatal mortality rate. The Gini coefficient of obstetric care facilities had a slightly stronger influence on the perinatal mortality rate than the number of obstetricians. The perinatal mortality rate was not observed to be significantly related to either the number of obstetric care facilities or the Gini coefficient of the obstetricians.

## 4. Discussion

There was a negative relation between the perinatal mortality rate and the number of obstetricians and the Gini coefficient of obstetric care facilities. However, no significant correlation was observed between the perinatal mortality rate and the number of obstetric care facilities. The number of obstetricians might be more influential on the neonatal mortality rate compared to the number of obstetric care facilities. We propose that increasing the number of obstetricians can lower the perinatal mortality rate. A negative correlation between the number of obstetricians/gynecologists and early neonatal mortality rate has also been demonstrated in a previous study [27]. When the number of obstetricians is reduced, perinatal mortality rate certainly worsens; therefore, it is important to retain the number of obstetricians. Improvements in the working environment and offering economic incentives are conceivable measures for increasing the number of obstetricians.

The centralization of obstetric care facilities (increase in Gini coefficient) has been suggested as a suitable approach to lower the perinatal mortality rate. The Gini coefficient of obstetric care facilities had a slightly stronger effect on the perinatal mortality rate than the number of obstetricians. We suggest that centralizing obstetric care facilities can improve the perinatal mortality rate even when it is difficult to increase the number of obstetricians. Centralization is expected to result in an improved working environment. There is a possibility that centralization can improve the safety of medical care facilities. Possible measures for further centralization include improving emergency transport methods; applying information technology; standardizing medical care; and using midwives. However, it would be skeptical to assume that further centralization will contribute to maintaining safety with regard to pregnancy and delivery. Buchmueller in his study [6] found that excessive centralization worsened perinatal mortality rate.

The first potential limitation of the present study is that we were unable to evaluate access to medical care. The degree of centralization of obstetricians and obstetric care facilities may not necessarily be related to access to medical care. Secondly, we were unable to categorize obstetric care facilities by the levels of care they provide. In the future, it will be necessary to measure changes over time while accounting for access to medical care. Lastly, because this study was officially announced and that everyone could access the data, it is also important and worth analyzing the effects of other factors on perinatal mortality rate in future.

## 5. Conclusion

We demonstrated that increasing the number of obstetricians and centralizing obstetric care facilities can improve the perinatal mortality rate. We also proved that centralizing obstetric care facilities can improve the perinatal mortality rate, even when increasing the number of obstetricians is difficult.

## Figures and Tables

**Table 1:** Perinatal mortality rate, total fertility rate, Gini coefficients, and obstetric care resources by prefecture.

	Perinatal mortality rate	Total fertility rate	Gini coefficient of obstetric care facilities (per capita)	Gini coefficient of obstetricians (per capita)	Number of obstetric care facilities (per capita)	Number of obstetricians (per capita)
Hokkaido	4.4	1.19	0.185	0.272	1.852	4.979
Aomori	4.8	1.26	0.130	0.284	2.258	5.731
Iwate	5.4	1.37	0.104	0.197	3.232	6.246
Miyagi	3.9	1.25	0.201	0.232	2.002	5.187
Akita	4.7	1.29	0.228	0.146	2.579	5.525
Yamagata	4.2	1.39	0.057	0.081	2.995	6.066
Fukushima	4.9	1.49	0.252	0.253	2.612	5.028
Ibaragi	3.9	1.37	0.156	0.211	2.088	5.143
Tochigi	4.1	1.43	0.114	0.222	2.142	7.583
Gunma	5.0	1.38	0.175	0.204	2.390	4.830
Saitama	3.9	1.28	0.170	0.161	1.487	4.575
Chiba	5.1	1.31	0.100	0.191	1.850	5.581
Tokyo	3.9	1.12	0.303	0.226	1.451	6.112
Kanagawa	4.8	1.28	0.066	0.131	1.337	5.112
Niigata	4.0	1.37	0.114	0.076	2.105	5.272
Toyama	5.1	1.37	0.054	0.093	2.378	7.033
Ishikawa	3.8	1.40	0.081	0.099	2.991	6.248
Fukui	2.8	1.55	0.283	0.439	2.728	7.403
Yamanashi	4.4	1.31	0.382	0.470	1.739	5.865
Nagano	3.7	1.43	0.220	0.230	2.137	5.286
Gifu	4.8	1.37	0.224	0.248	2.691	5.319
Shizuoka	3.4	1.43	0.109	0.182	1.992	4.900
Aichi	4.4	1.43	0.229	0.312	2.038	6.293
Mie	3.4	1.40	0.162	0.130	2.157	5.747
Shiga	4.0	1.44	0.164	0.234	2.907	4.864
Kyoto	3.6	1.20	0.207	0.180	2.427	6.906
Osaka	3.9	1.28	0.082	0.135	1.771	5.966
Hyogo	4.0	1.33	0.129	0.109	2.075	5.353
Nara	5.2	1.23	0.158	0.321	1.929	5.279
Wakayama	5.2	1.36	0.246	0.160	2.397	5.693
Tottori	4.1	1.46	0.051	0.205	3.229	7.971
Shimane	4.6	1.55	0.108	0.232	3.071	7.678
Okayama	4.1	1.39	0.114	0.145	2.365	6.309
Hiroshima	4.4	1.47	0.138	0.111	2.342	5.764
Yamaguchi	4.1	1.43	0.106	0.113	2.756	6.408
Tokushima	4.1	1.35	0.290	0.513	3.054	6.070
Kagawa	3.5	1.48	0.067	0.217	2.611	6.347
Ehime	4.7	1.41	0.201	0.199	2.656	5.968
Kochi	3.3	1.29	0.131	0.300	2.747	6.278
Fukuoka	4.0	1.37	0.167	0.219	2.563	6.099
Saga	3.2	1.49	0.143	0.172	3.648	6.908
Nagasaki	4.3	1.50	0.151	0.162	3.785	6.870
Kumamoto	3.5	1.58	0.249	0.297	3.026	6.784
Oita	4.6	1.50	0.159	0.091	2.675	4.263
Miyazaki	3.6	1.61	0.245	0.280	3.788	7.691
Kagoshima	4.0	1.56	0.137	0.223	3.282	6.815
Okinawa	4.8	1.79	0.136	0.218	2.729	7.655

Mean (SD)	5.387 (0.510)	1.395 (0.122)	0.163 (0.073)	0.211 (0.095)	2.491 (0.585)	6.021 (0.914)

**Table 2:** Regression results for the perinatal mortality rate and obstetric care resources.

	Non-standardized coefficient	Standardized coefficient	*t*	*P*
Total fertility rate	−1.126	−0.270	−1.550	0.129
Number of obstetricians	−0.240	−0.430	−2.536	0.015
Number of obstetric care facilities	0.290	0.333	1.831	0.074
Gini coefficient of obstetricians	1.187	0.220	1.178	0.246
Gini coefficient of obstetric care facilities	−3.391	−0.485	−2.620	0.012

*R* ^2^ = 0.286.

Adjusted *R* ^2^ = 0.198.
